# A selective and label-free strategy for rapid screening of telomere-binding Ligands via fluorescence regulation of DNA/silver nanocluster

**DOI:** 10.1038/srep42629

**Published:** 2017-03-06

**Authors:** Rui Cheng, Jing Xu, Xiafei Zhang, Zhilu Shi, Qi Zhang, Yan Jin

**Affiliations:** 1 Key Laboratory of Applied Surface and Colloid Chemistry, Ministry of Education, Key Laboratory of Analytical Chemistry for Life Science of Shaanxi Province, School of Chemistry and Chemical Engineering, Shaanxi Normal University, Xi’an 710062, China

## Abstract

Herein, the conformational switch of G-rich oligonucleotide (GDNA) demonstrated the obvious functional switch of GDNA which was found to significantly affect the fluorescence of the in-situ synthesized DNA/silver nanocluster (DNA-AgNC) in homogeneous solution. We envisioned that the allosteric interaction between GDNA and DNA-AgNC would be possible to be used for screening telomere-binding ligands. A unimolecular probe (12C5TG) is ingeniously designed consisting of three contiguous DNA elements: G-rich telomeric DNA (GDNA) as molecular recognition sequence, T-rich DNA as linker and C-rich DNA as template of DNA-AgNC. The quantum yield and stability of 12C5TG-AgNC is greatly improved because the nearby deoxyguanosines tended to protect DNA/AgNC against oxidation. However, in the presence of ligands, the formation of G-quadruplex obviously quenched the fluorescence of DNA-AgNC. By taking full advantage of intramolecular allosteric effect, telomere-binding ligands were selectively and label-free screened by using deoxyguanines and G-quadruplex as natural fluorescence enhancer and quencher of DNA-AgNC respectively. Therefore, the functional switching of G-rich structure offers a cost-effective, facile and reliable way to screen drugs, which holds a great potential in bioanalysis as well.

The conformational transition of G-rich oligonucleotide in response to specific stimuli is close related to various biological processes. Under physiological condition, G-rich oligonucleotide can assemble into different structures, including hairpins, cruciforms, triplexes and G-quadruplexes etc, which might be involved in many important biological events^1^. Based on those, the development of G-quadruplex for bio/nano technology is attracted considerable attentions. For instance, G-rich oligonucleotide folds into the G-quadruplex-hemin structure in the presence of K^+^ and hemin, exhibiting horseradish peroxidase mimicking functions^2^,^3^,^4^,^5^,^6^,^7^,^8^. Compared to peroxidases, G-quadruplex-Hemin DNAzyme is smaller in size, easier to functionalize, cheaper to prepare, and more stable under harsh conditions and can undergo many cycles of denature/renature, attracting more attention in bioanalysis. Moreover, G-quadruplexes have also demonstrated great potentials as therapeutic drug targets. It is well known that the telomere length can be maintained to induce cellular immortalization due to the activation of telomerase in cancer cells^9^,^10^. Researches suggested that G-quadruplex formation at the 3′ end of telomere DNA could directly block its binding with telomerase and inhibit telomere extension *in vitro*^11^,^12^. A variety of methods have been reported for exploring the formation and properties of G-quadruplexes, especially for discovering G-quadruplex-binding ligands. To achieve facile and reliable screening of G-quadruplex-binding ligands, we have developed fluorescent and electrochemical strategies for rapidly discovering G-quadruplex-targeted binding ligands^13^,^14^,^15^,^16^,^17^,^18^. Although the above methods have made progress in discovery of G-quadruplex ligands, the lack of high-throughput and label-free method still limited the discovery of potent telomerase inhibitors from the large chemical libraries of candidates.

Herein, based on the functional switch of G-rich DNA (GDNA) on the DNA-AgNC, a specific and label-free strategy is presented for rapid screening of telomere-binding lignds. DNA-AgNC has been proved to be of facile synthesis, tunable fluorescence emission, and high photostability^19^,^20^,^21^ and has been applied in bioimaging^22^, catalysis^23^,^24^, and chemical sensing^25^,^26^. The photophysical properties of DNA-AgNC are largely dependent on the specific sequences and structures of DNA templates^27^,^28^. Werner’s group have discussed that weak fluorescence of DNA-templated AgNC could be strongly enhanced when AgNC was in proximity to G-rich sequences^29^. The recent report also demonstrated that the fluorescence of AgNC changed as the single-stranded DNA converted into quadruplex structure^30^. We envisioned that the allosteric interaction of GDNA and G-quadruplex on the photophysical properties of DNA-AgNC would be possible to be utilized for screening telomere-binding ligands. A unimolecular probe (12C5TG) is ingeniously designed consisting of three contiguous DNA elements: G-rich telomereic DNA (GDNA) as molecular recognition sequence, T-rich DNA as linker and C-rich DNA as template of DNA-AgNC. Initially, the fluorescence of DNA/AgNC was enhanced by G-rich single-stranded DNA. In the presence of ligands, G-quadruplex formation effectively quenched the fluorescence of DNA/AgNC. Based on the fluorescence changes of in-situ synthesized DNA-AgNC, G-quadruplex-binding ligand could be conveniently and specifically identified. To our best knowledge, this is the first approach to screening of G-quadruplex ligands by fluorescence regulation of DNA-AgNC.

## Results and Discussion

### Synthesis and Characterization of DNA-AgNC

**Figure 1:**
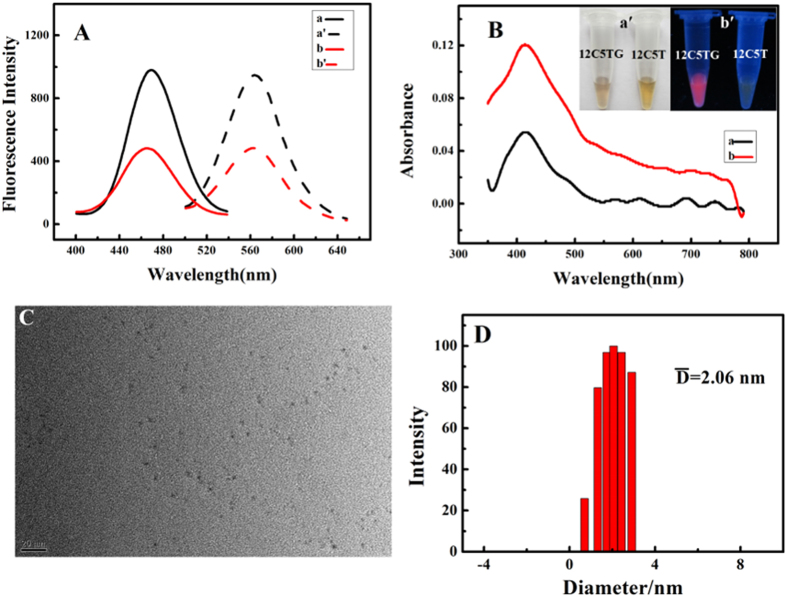
Characterization of DNA-AgNC. (**A**) Fluorescence excitation/emission spectra of (a/a′) 1.0 μM 12C5TG-AgNC and (b/b′) 12C5T-AgNC. (**B**) UV-vis absorption spectra of (a) 10.0 μM 12C5T-AgNC (b) 12C5TG-AgNC. Inset: pictures of the AgNC solution under (a′) room lighting and (b′) UV irradiation. (**C**) TEM image of 5.0 μM 12C5TG-AgNC. The bar is 20 nm scales. (**D**) DLS characterization of the prepared 12C5TG-AgNC.

Generally, most DNA/Ag nanocluster was synthesized via the reduction of a mixture of DNA scaffold and silver nitrate by sodium borohydride. It was also reported that visible and near-IR-emitting AgNC could be generated through single-stranded DNA (ssDNA) template containing 12 cytosines^21^. To verify the enhancement effect of G-rich DNA, a DNA template (12C5T) without the human telomeric repeats was designed as a negative control. The luminescence properties of DNA-AgNC have been investigated by fluorescence (Fig. 1A) and UV-vis absorption spectra (Fig. 1B) measurements. In Fig. 1A, 12C5TG-stabilized AgNC (12C5TG-AgNC) showed a maximum emission at 564 nm and a maximum excitation at 470 nm and its fluorescence intensity was nearly 1-fold stronger than that of 12C5T-templated AgNC (12C5T-AgNC) because DNA-AgNC was in proximity to G-rich sequences^29^. In Fig. 1B, the UV-Vis absorbance of 12C5TG-AgNC at 470 nm was 1-fold higher than that of 12C5T-AgNC, which was consistent with the fluorescence result. Photographs of the DNA-AgNC were displayed in the inset of Fig. 1B. Both the 12C5TG-AgNC and 12C5T-AgNC present the color of yellow under the irradiation of room-light. Upon UV irradiation, 12C5TG-AgNC shows red, while 12C5T-AgNC shows green. This phenomenon was explained by James H. Werner as follows^29^. The nearby deoxyguanosines tended to protect Ag nanoclusters against oxidation by serving as an electron donor and a reducing agent. So, the reduced AgNC showed a red-emitting. Conversely, Ag nanocluster without deoxyguanosines protection can be oxidized in a few hours in air-saturated solutions, showing a green-emitting of oxidized species. These results indicated that the fluorescence of 12C5TG-AgNC was enhanced and stabilized by the nearby guansines. Transmission electron microscopy image in Fig. 1C revealed that the 12C5TG-AgNC is monodispersed and the average diameter in the distribution is around 2.06 nm shown in Fig. 1D. The fluorescence quantum yield of 12C5TG-AgNC is calculated to be 40.0% when Rhodamine 6 G in ethanol is used as the reference standard by assuming its luminescence quantum yield as 95% at room temperature (Figure S1)^31^. All results discussed above demonstrated the DNA-AgNC has been successfully prepared for further use.

### Proof of Principle

**Figure 2:**
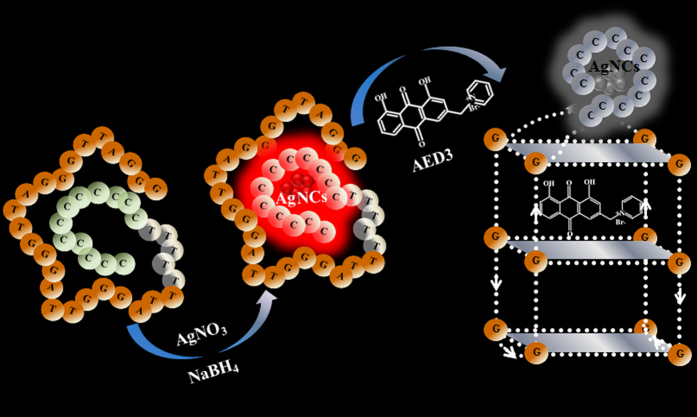
Schematic illustration of identification of telomere-binding ligands via fluorescence regulation of DNA/silver nanocluster.

G-quadruplex formation is one of the effective pathways for inhibiting telomerase activity because G-quadruplex formation at the 3′ end of telomere DNA could directly block its binding with telomerase and inhibit telomere extension *in vitro*. In this work, a label-free fluorescence strategy has been developed to identify G-quadruplex-binding ligands by in-situ synthesis of DNA/Ag nanocluster as signal transducer. As shown in Fig. 2, an allosteric unimolecular probe was ingeniously designed. This unimolecular probe includes three main components. Its 3′ end is human telomeric DNA (GDNA) which serves as functional switching domain as well as molecular recognition domain. The sequence of 12 cytosines at 5′ end of the unimolecular probe acts as template sequence for in situ-synthesis of DNA-AgNC. To reduce steric hindrance, the G-rich domain links C-rich domain via a T-rich linker. Initially, the single-stranded unimolecular probe (12C5TG) reacted with silver nitrate and sodium borohydride to form DNA-AgNC whose fluorescence can be enhanced by the adjacent deoxyguanosines. Then, when the probe captured its target, the formation of G-quadruplex disturbed the intramolecular conformation, and this event switched the function of GDNA from a fluorescence enhancer to a fluorescence quencher, which subsequently gives a turn-off fluorescence signal. Therefore, this design utilized the intramolecular allosteric effect to facilely and specifically screening of G-quadruplex ligands in homogeneous solution.

**Figure 3:**
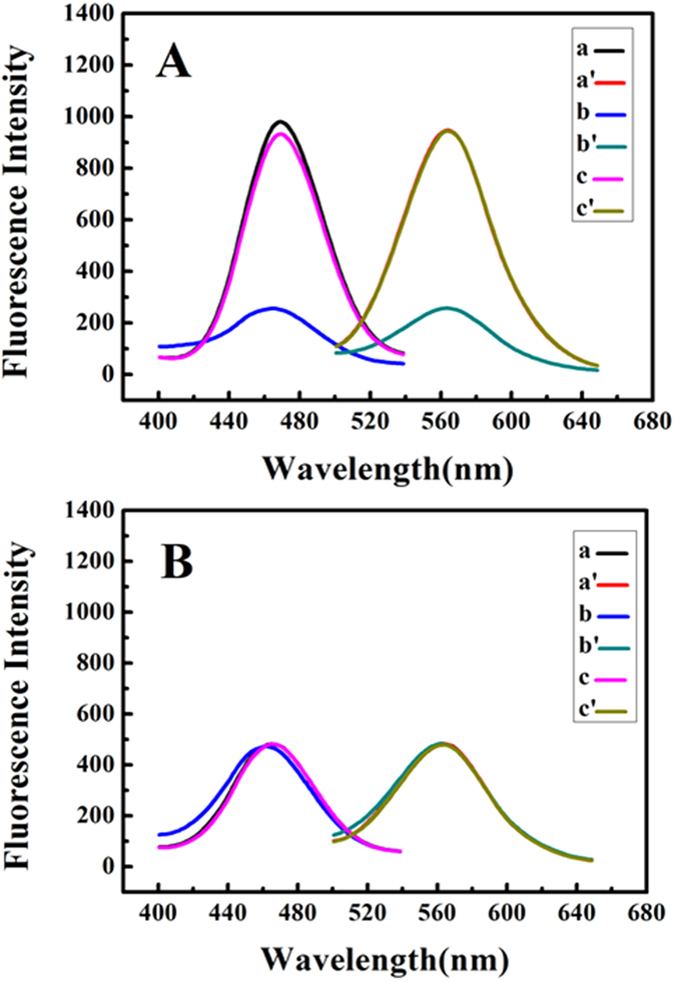
Fluorescence excitation/emission spectra of (**A**) (a/a′) 1.0 μM 12 C 5TG-AgNC; (b/b′) (a/a′) +10.0 μM AED3; (c/c′) (a/a′) +10.0 μM matrine; (**B**) (a/a′) 1.0 μM 12C5T-AgNC; (b/b′) (a/a′) +10.0 μM AED3; (c/c′) (a/a′) +10.0 μM matrine.

To test the feasibility of the proposed strategy, aloe-emodin derivative 3 (AED3) was chosen as a model ligand. The synthesis and characterization of AED3 have been described in our previously work, and AED3 shows superior planarity and good solubility (Figure S2 showed chemical structural formula of ligand)^32^. It is evident in Fig. 3A that the fluorescence of 12C5TG-AgNC is strong. In the presence of 10.0 μM AED3, the fluorescence intensity of 12C5TG-AgNC decreased ~72.9% (Fig. 3A curve b/b′). The interaction between human telomeric DNA and AED3 promoted the formation of G-quadruplex which can effectively quench the fluorescence of 12C5TG-AgNC. Therefore, the preliminary outcome proved the feasibility and effectiveness of this novel strategy.

Then, more control experiments were performed to verify the specificity of signal transduction. Matrine was chosen as a negative model molecule since it is not a G-quadruplex ligand according to our previous research^14^,^18^. The fluorescence intensity of 12C5TG-AgNC remained unchanged when they incubated with 10.0 μM matrine (Fig. 3A curve c/c′), indicating that negative ligand cannot cause such a change in fluorescence of 12C5TG-AgNC. To further exclude the possibility of fluorescence quenching induced by AED3, a control DNA template, 12C5T, was employed. As shown in Fig. 3B, the fluorescence intensity of 12C5T-AgNC was much lower than that of 12C5TG-AgNC due to the lack of guanosines. It is explicit that the fluorescence of 12C5T-AgNC is unchanged in the presence of AED3 or matrine. Therefore, AED3 itself will not affect the fluorescence of DNA-AgNC. All of these control experiments strongly indicated that the decrease in fluorescence of 12C5TG-AgNC is caused by the formation of G-quadruplex in the presence of AED3. To sum up, telomere-binding ligands can be effectively identified based-on the different influences of guanines and G-quadruplex on the fluorescence of DNA-AgNC.

### Identification of G-quadruplex Formation

**Figure 4:**
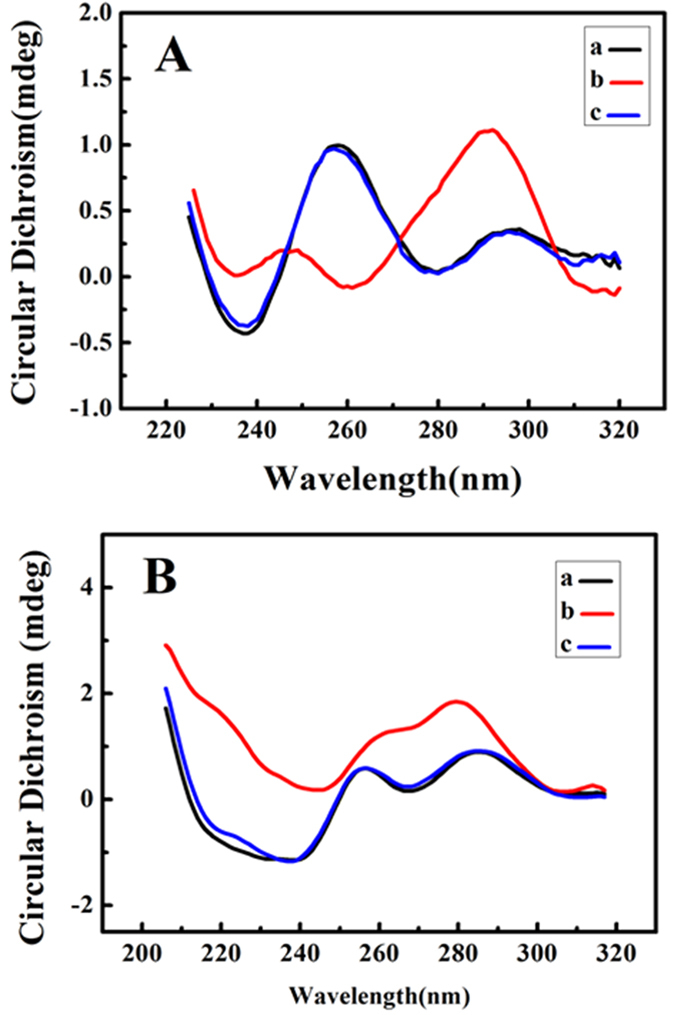
Circular dichroism spectroscopy of (**A**) (a) 2.0 μM GDNA; (b) (a) +20.0 μM AED3; (c) (a) +20.0 μM matrine; (**B**) (a) 2.0 μM 12C5TG-AgNC; (b) (a) +20.0 μM AED3; (c) (a) +20.0 μM matrine.

Circular dichroism (CD) is a sensitive and readily available method to judge the conformational conversion of DNA. It can be seen from Fig. 4A that the CD spectra of human telomeric DNA (GDNA) shows a positive peak around 258 nm and a negative peak near 237 nm (Fig. 4A curve a). In the presence of AED3, the negative peak near 261 nm and the positive peak around 292 nm appeared, indicating the formation of antiparallel G-quadruplexes (Fig. 4A curve b)^32^. However, the conformation of GDNA is unchanged when GDNA incubated with matrine (Fig. 4A curve c). These results suggested that single-stranded GDNA can fold into G-quadruplex in the presence of AED3. With AED3 (Fig. 4B curve b), the CD spectra of 12C5TG-AgNC also changed with two positive peaks near 262 nm and 280 nm, which illustrated the formation of the hybrid “antiparallel” and “parallel” G-quadruplexes^32^. By contrast, the presence of matrine (Fig. 4B curve c) led to few structural changes of 12C5TG-AgNC. Meanwhile, neither AED3 nor matrine could alter the structure of 12C5T-AgNC (Figure S3). It has been previously reported that Ag^+^ preferentially bonded with the bases and significantly affected DNA conformation, as evidenced by the development of two strong peaks with negative ellipticity around 215 and 265 nm and by the decreased ellipticity of the peak at 290 nm^33^,^34^. Therefore, compared with the G-quadruplex of GDNA4, the CD peaks of 12C5TG-AgNC induced by AED3 were slightly different, which were susceptible to be interfered by peaks of residual Ag^+^ and DNA. Thus it could not be pure “antiparallel” G-quruplex peaks. The results verified that AED3 can effectively induce G-rich human telomeric DNA to form G-quadruplex.

### Effect of External Factors

Changes in micro-environment would apparently affect the luminescence property of DNA-AgNC. To obtain DNA-AgNC with strong fluorescence, several parameters, such as pH, the number of cytosine and the length of linker T, were optimized. Firstly, three commonly used buffer were compared^33^,^34^,^35^. As shown in Figure S4, 10 mM Tris-Ac buffer (pH 8.0) was selected as the final medium. Then, the effect of pH on the fluorescence quenching efficiency (F_0_ - F)/F_0_ of 12C5TG-AgNC was investigated (Figure S5), where F_0_ and F were the fluorescence intensity of the 1.0 μM 12C5TG-AgNC in the absence and presence of 10.0 μM AED3, respectively. Taking into account (F_0_ - F)/F_0_ and physiological condition, pH 8.0 was chosen as the optimal pH. Furthermore, a series of DNA templates with different number of cytosines were utilized for synthesis of DNA-AgNC (Figure S6). All of the DNA sequences are listed in Table S1. The results indicated twelve cytosines is the best template. Lastly, the effect of linker lengths was also examined (Figure S7). The fluorescence intensity of DNA-AgNC enhanced with the increasing of linker lengths and reached to a maximum when the number of thymine exceeds five. The longer T loop facilitated the fold of the guanine-rich closely to AgNC. However, as the number of thymine increased, the fluorescence quenching efficiency (F_0_ - F)/F_0_ with AED3 decreased when T number was more than five. This result may be attributed to the long distance between DNA-AgNC and G-quadruplex. Therefore, five thymines were used as linker when taken all associated factors into account.

### Identification of G-quadruplex-binding Ligands

**Figure 5:**
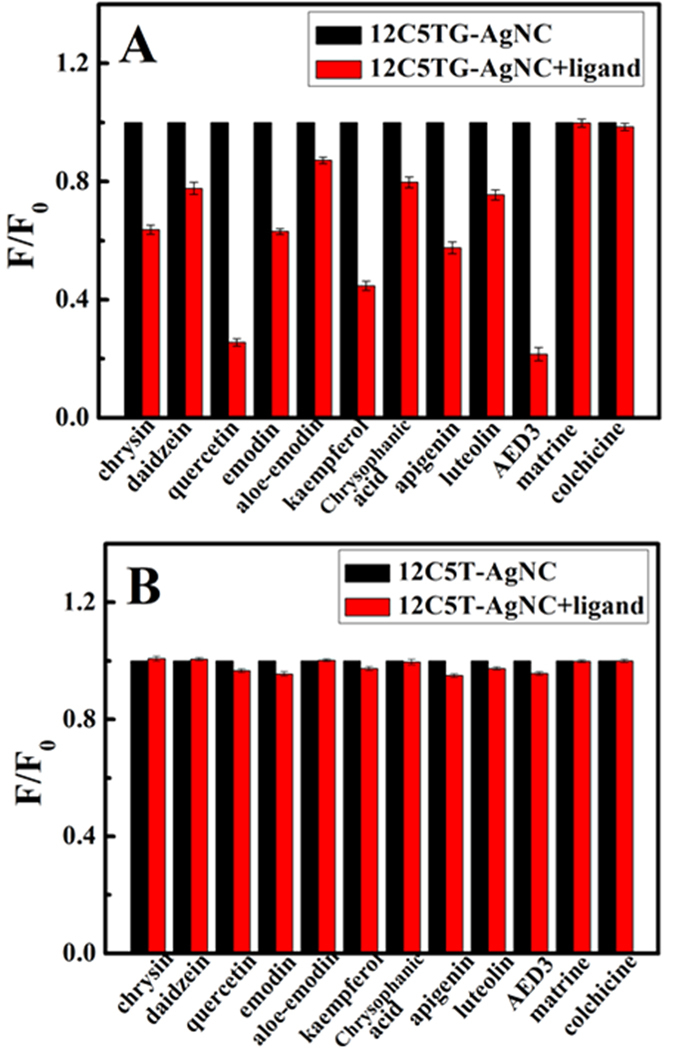
Fluorescence quenching efficiency of (**A**) 1.0 μM 12C5TG-AgNC with 20.0 μM ligands and (**B**) 1.0 μM 12C5T-AgNC with 20.0 μM ligands, where F_0_ and F were the fluorescence intensity of AgNC in the absence and presence of ligand, respectively.

G-quadruplex formation can effectively inhibit telomerase activity in most cancer cells by locking the single-stranded telomeric substrate into an inactive conformationm, which is neither recognized nor elongated by telomerase. So, screening of G-quadruplex ligands is meaningful for the discovery of telomerase-targeted anticancer drugs. In this paper, three kinds of anticancer drug monomers with antiblastic activity were inspected. Aloe-emodin, aloe-emodin derivative 3 (AED3), chrysophanic acid and emodin belong to anthraquinones. The second type is flavonoids, including daidzein, quercetin, luteolin, chrysin, apigenin and kaempferol. Colchicine and matrine belong to the alkaloids. The impact of twelve drug monomers on the fluorescence response of 12C5TG-AgNC was demonstrated by comparing quenching ratio (F/F_0_) in Fig. 5A, where F_0_ and F were the fluorescence intensity of the 1.0 μM 12C5TG-AgNC in the absence and presence of 20.0 μM ligands, respectively. It is clear that chrysin, daidzein, quercetin, emodin, aloe-emodin, kaempferol, chrysophanic acid, apigenin, luteolin and AED3 induced strong fluorescence quenching of 12C5TG-AgNC, indicating that they are G-quadruplex-interactive ligands. On the contrary, both colchicine and matrine are not G-quadruplex ligands because they were unable to induce the changes in the fluorescence of 12C5TG-AgNC. In order to exclude the possibility of fluorescence quenching by ligands themself, 12C5T-AgNC was incubated with different ligands. Figure 5B indicated that the fluorescence of 12C5T-AgNC was unchanged in the presence of all ligands. Therefore, the binding between ligand and GDNA was the essential factor for inducing fluorescence quenching of 12C5TG-AgNC. To further verify the reliability, the inhibition effect of the selected ligands on the telomerase activity had been studied by telomeric repeat amplification protocol (TRAP) which is a landmark method for telomerase activity detection. It is evident from Figure S8 that telomerase activity can be effectively inhibited by the selected ligands. Conversely, in the presence of matrine and colchicine, the activity of telomerase barely changed. The TRAP data indicated that it is a reliable method for screening G-quadruplex-binding ligands.

### Inhibition Effect of Ligands on the Growth of Cancer Cell

**Figure 6:**
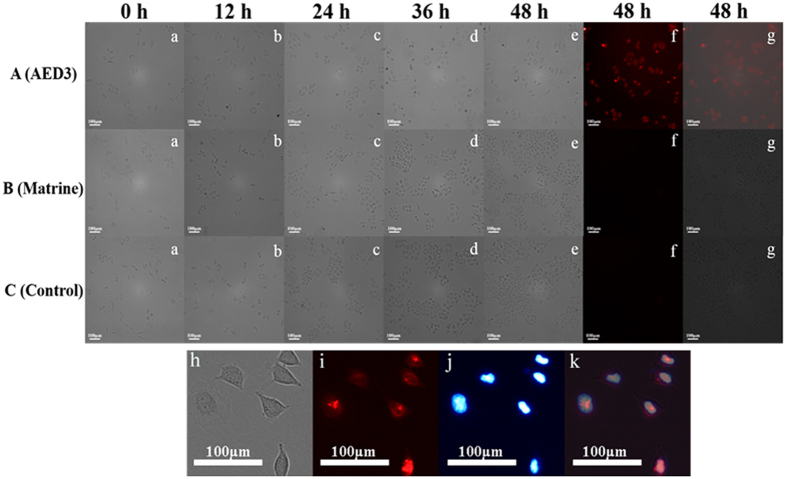
Time course of images of HeLa incubated with 25 μM AED3 (**A**), 25 μM matrine (**B**) and blank control (**C**). (a–e) bright-field images, (f) fluorescence image and (g) the overlay of the fluorescence image and the bright-field image. Images h-k represented for intracellular distribution of AED3 after 48 h. (h) bright-field image, (i) fluorescence image of AED3 (red), (j) fluorescence image with Hoechst 33342 nuclear staining (blue), (k) overlap of corresponding fluorescence image and bright-field image.

G-quadruplex formation at the end of telomeric DNA is one of the pathways to inhibit the activity of telomerase^11^,^12^. The inhibition effect of the selected G-quadruplex binding ligand (e.g. AED3) on human cervical cancer (HeLa) cells was investigated using imaging technology. As shown in Fig. 6, cell density increased with the increasing of the incubating time from 0 h to 48 h in both matrine group (a–e) and control (a–e) group, suggesting that matrine can’t inhibit proliferation of tumour cells. On the contrary, no obvious increase in the cell density of AED3 group (a–e) has been observed within same incubation time, which was attributed to efficient inhibition capability of AED3. From fluorescence imaging, a bright and red fluorescence emission of AED3 was obtained and occurred around and within the nucleus. Under the same procedure, there was no detectable fluorescence from the control and matrine group. By comparing bright-field image with nuclear Hoechst staining, we found the intracellular AED3 was mainly accumulated in cytoplasm and especially in the nuclei of HeLa cells (Fig. 6(h–k)) since AED3 has favorable planarity and positive charge, which facilitate cellular uptake of AED3 without the carrier. The inhibition effect of the other ligands also had been studied. All the selected ligands can inhibit the growth of cancer cells. The related data was supplemented in Figure S9.

MTT assay has been widely used in the large-scale anti-tumor drugs screening and cell toxicity test. The *in vitro* cytotoxicity of the selected ligands toward HeLa and Acute lymphoblastic leukemia T-cells (CCRF-CEM) was estimated via MTT assay. After incubating HeLa cells with different concentrations of AED3 for 48 h, the absorbance was measured to calculate the relative cell viability at each concentration. After treated with AED3 of 3.92 μM for 48 h, the HeLa cells maintained about 50% of the cell viability (Figure S10). CCRF-CEM after AED3 treatment showed a half-maximal inhibitory concentration (IC_50_) value of 5.98 μM. Because the number of HeLa cell was less than that of CCRF-CEM cell for MTT assay, AED3 inhibited the former more strongly than the later at same concentration as the histogram analysis. MTT photograph (Figure S10B) corresponded to the result of Figure S10A. The IC_50_ value of other ligands towards HeLa is listed in Table S2 suggesting AED3 has stronger inhibitory ability than others, which shows a consistent phenomenon with the fluorescent result (Fig. 5A). It once again verifies our screening method is highly reliable.

## Conclusion

In summary, DNA-AgNC was firstly explored as fluorescence probe to establish a reliable and facile platform for screening G-quadruplex-binding ligands. It makes full use of the functional switching of guanine-rich telomeric DNA on the fluorescenc of DNA-AgNC to accomplish specific signal conversion. DNA-AgNC with high quantum yield was easily in-situ synthesized without any separation and other tedious process. Telomere-binding ligands could be rapidly and label-freely screened. This study provided a new bioanalysis platform via functional switching of G-rich DNA on the property of DNA nanocluster.

## Methods

### Chemicals

The DNA sequences (listed in Table S1 in surpporting information) were synthesized by Sangon Biotech Co., Ltd. (Shanghai, China). Silver nitrate (99.99%) was purchased from Alfa-Aesar (Tianjin, China). Both sodium borohydride (98%) and rhodamine 6 G (98.5%) were obtained from J&K^®^ (Beijing, China). Traditional chinese medicine monomer, such as chrysin, daidzein, quercetin, emodin, aloe-emodin, kaempferol, chrysophanic acid, apigenin, luteolin, matrine and colchicine were purchased from Nanjing TCM Institute of Chinese Material Medical (Nanjing, China). Aloe-emodin derivative (AED3) was synthesized by our own group and chemical structural formulas of all drugs are listed in Figure S1 (Supporting Information). Dulbecco’s modified Eagle’s medium (DMEM, GIBCO), RPMI-1640 and phosphate buffer saline (PBS, pH 7.4) were purchased from HyClone Thermofisher (Beijing, China). Drugs and other reagents were of analytical reagent grade and used without further purification. The oligonucleotide stock solutions were prepared with 10 mM Tris-acetic acid (Tris-Ac) buffer (pH 8.0). All aqueous solutions were freshly prepared in ultrapure water (≥18MΩ, Milli-Q, Millipore). HeLa cells and CCRF-CEM cells were purchased from KeyGen Biotech. Co. Ltd. (Nanjing, China), AGS cells and HL-7702 cells were purchased from Cell Bank of Chinese Academy of Science (Shanghai, China).

### Preparation of Fluorescent DNA/Ag Nanocluster

Oligonucleotide stabilized AgNC were synthesized according to the procedure described as previously reported^28^. Briefly, 20 μM 12C5TG and 120 μM AgNO_3_ were premixed in 10 mM Tris-Ac buffer (pH 8.0) to reach a nucleobase to Ag^+^ molar ratio of 1:6. After being chilled on ice for 15 min, the mixture was reduced by quickly adding 120 μM NaBH_4_, followed by vigorous shaking for 1 min. The reaction was kept in the dark at 4 °C for at least 4 h before use. AgNC stabilized with other DNA sequences listed in Table S1 were synthesized by similar procedure.

### Characterization

Typically, the UV absorption spectra of both DNA 12C5TG and DNA 12C5T (listed in Table S1) templated AgNC (12C5TG-AgNC, 12C5T-AgNC) were recorded on UV-vis spectrophotometer (Hitachi U-3900H) and their photographs under room-light and UV irradiation were taken by a digital camera. TEM images were obtained by using a JEM-2100 transmission electron microscope. For the determination of quantum yield, UV-vis spectra was measured from 350 to 800 nm at a scanning speed of 300 nm/min. Fluorescence emission spectra of the standard Rhodamine 6 G and 12C5TG-AgNC were obtained with excitation at 498 nm, where the absorbance values of them are equal. The quantum yield of the AgNC was calculated using the following equation, with reference to Rhodamine 6 G^31^


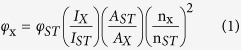


where the subscripts ST and X denote standard and test respectively, φ_x_ is the fluorescence quantum yield of AgNC and φ_ST_ is 95% (ethanol)^33^,^36^. I is the integrated fluorescence emission peak areas, A_X_ and A_ST_ are equal in this case, which Rhodamine 6 G is the absorption at the specific wavelength used for fluorescence excitation, n_X_ (H_2_O, 1.3329) and n_ST_ (ethanol, 1.3611) are the refractive indices of solvents.

### Fluorescence Measurements

Fluorescence measurements were performed on a Hitachi F-7000 fluorescence spectrophotometer (Tokyo, Japan) and the excitation/emission wavelengths were set at 470 nm/564 nm. A portion of 20 μM 12C5TG-AgNC was diluted with 10 mM Tris-Ac buffer (pH 8.0). After ligand solution has been added into the above reaction system for 5 mins, the fluorescence was measured to obtain its excitation and emission spectra with a final volume of 200 μL. The detection procedure of AgNC stabilized by other DNA sequences listed in Table S1 was the same as those described in the aforementioned experiment for 12C5TG-AgNC detection.

### Circular Dichroism Spectroscopy

A Chirascan Circular Dichroism Spectrometer (Applied Photophysics Ltd, England, UK) was utilized to collect the CD spectra of sample (2 μM). CD spectra were performed using an optical chamber (1 mm optical path length) with an instrument scanning speed of 100 nm/min, a response time of 2 s at room temperature and were accumulated by taking the average of three scans made from 200 to 320 nm.

### Cell Culture

Human cervical carcinoma (HeLa) and acute lymphoblastic leukemia T-cells (CCRF-CEM) were cultured in DMEM and RPMI-1640 medium, respectively, supplemented with 10% fetal calf serum (FCS, Sigma), penicillin (100 μg/mL), and streptomycin (100 μg/mL) at 37 °C in a humidified chamber, containing 5% CO_2_.

### Fluorescence Microscopy Imaging

After most HeLa cells adherently grow onto the petri dish, fresh culture medium containing drugs (25 μM) were added into the specimen for a certain incubating time at 37 °C in a humidified chamber. After cells were washed three times with PBS, imaging of bright field was performed on an Olympus IX73 microscope (Olympus, Japan) with a 10× objective. The fluorescence imaging of specimens incubated with drugs for 48 h was excited with green channel.

### MTT Assay

The inhibition efficiency of ligands was tested with HeLa cells and CCRF-CEM cells by MTT assay. After cells were incubated with 200 μL culture medium containing different concentrations of ligands for 48 h, MTT (20 μL, 5 mg mL^−1^) was added into the well and incubated at 37 °C for 4 h. Then 150 μL of dimethyl sulphoxide was added to each well and shaken for 15 min to dissolve the crystals formed by the living cells, and the absorbance at 490 nm was measured to obtain the relative cell viability (%) by (A_test_/A_control_) × 100%. MTT assay was performed on a microplate reader (Thermo Fisher, Thermo Scientific Multiskan GO). Cell number was determined using a Petroff-Hausser cell counter. The density of HeLa cell and CCRF-CEM cell for MTT assay was 2 × 10^3^ cell/well and 2.4 × 10^4^ cell/well, respectively.

## Additional Information

**How to cite this article**: Cheng, R. *et al*. A selective and label-free strategy for rapid screening of telomere-binding Ligands via fluorescence regulation of DNA/silver nanocluster. *Sci. Rep.* **7**, 42629; doi: 10.1038/srep42629 (2017).

**Publisher's note:** Springer Nature remains neutral with regard to jurisdictional claims in published maps and institutional affiliations.

## Supplementary Material

Supplementary Information
