# Supplementary material for: A selective and label-free strategy for rapid screening of telomere-binding Ligands via fluorescence regulation of DNA/silver nanocluster
Source: Sci Rep. 2017 Mar 6;7:42629. doi: 10.1038/srep42629 (PMC5338008; doi:10.1038/srep42629)
Supplement: Supplementary Information [file srep42629-s1.doc]

**Supporting Information**

**A selective and label-free strategy for rapid screening of telomere-binding Ligands via fluorescence regulation of DNA/silver nanocluster**

Rui Cheng, Jing Xu, Xiafei Zhang, Zhilu Shi, Qi Zhang*, Yan Jin***

Key Laboratory of Applied Surface and Colloid Chemistry, Ministry of Education, Key Laboratory of Analytical Chemistry for Life Science of Shaanxi Province, School of Chemistry and Chemical Engineering, Shaanxi Normal University, Xi′an 710062, China

* Corresponding Author: [jinyan@snnu.edu.cn](mailto:jinyan@snnu.edu.cn), [qiqizhang@snnu.edu.cn](mailto:qiqizhang@snnu.edu.cn), Fax: 86-29-81530727, Tel: 86-29-81530726

**Table S1**. Oligonucleotides used in this study.

| **Name** | **Sequence(5′-3′)** |
| --- | --- |
| **18C5TG** | **CCCCCCCCCCCCCCCCCC*TTTTT*TTAGGGTTAGGGTTAGGGTTAGGG** |
| **15C5TG** | **CCCCCCCCCCCCCCC*TTTTT*TTAGGGTTAGGGTTAGGGTTAGGG** |
| **12C5TG** | **CCCCCCCCCCCC*TTTTT*TTAGGGTTAGGGTTAGGGTTAGGG** |
| **9C5TG** | **CCCCCCCCC*TTTTT*TTAGGGTTAGGGTTAGGGTTAGGG** |
| **6C5TG** | **CCCCCC*TTTTT*TTAGGGTTAGGGTTAGGGTTAGGG** |
| **3C5TG** | **CCC*TTTTT*TTAGGGTTAGGGTTAGGGTTAGGG** |
| **12C9TG** | **CCCCCCCCCCCC*TTTTTTTTT*TTAGGGTTAGGGTTAGGGTTAGGG** |
| **12C7TG** | **CCCCCCCCCCCC*TTTTTTT*TTAGGGTTAGGGTTAGGGTTAGGG** |
| **12C3TG** | **CCCCCCCCCCCC*TTT*TTAGGGTTAGGGTTAGGGTTAGGG** |
| **12CTG** | **CCCCCCCCCCCC*T*TTAGGGTTAGGGTTAGGGTTAGGG** |
| **12C5T** | **CCCCCCCCCCCC*TTTTT*** |
| **GDNA** | **TTAGGGTTAGGGTTAGGGTTAGGG** |

The red and blue letters were, respectively, representative for the cytosine-rich template sequence of DNA/Ag nanocluster and an oligonucleotide mimicking the human telomeric repeat. Italic letters were rich-thymine linker incorporated cytosine-rich sequence and the human telomeric repeat.


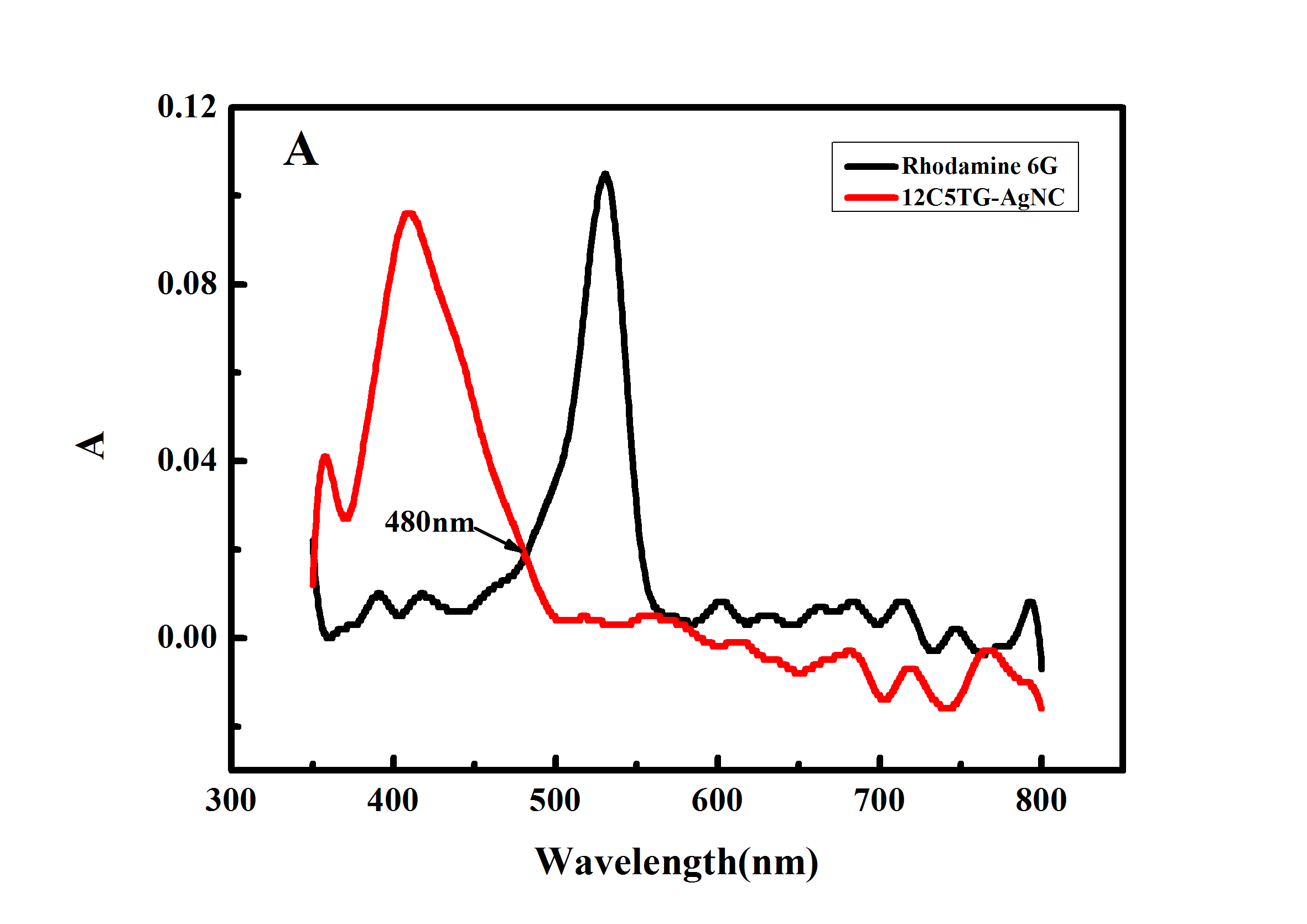


**
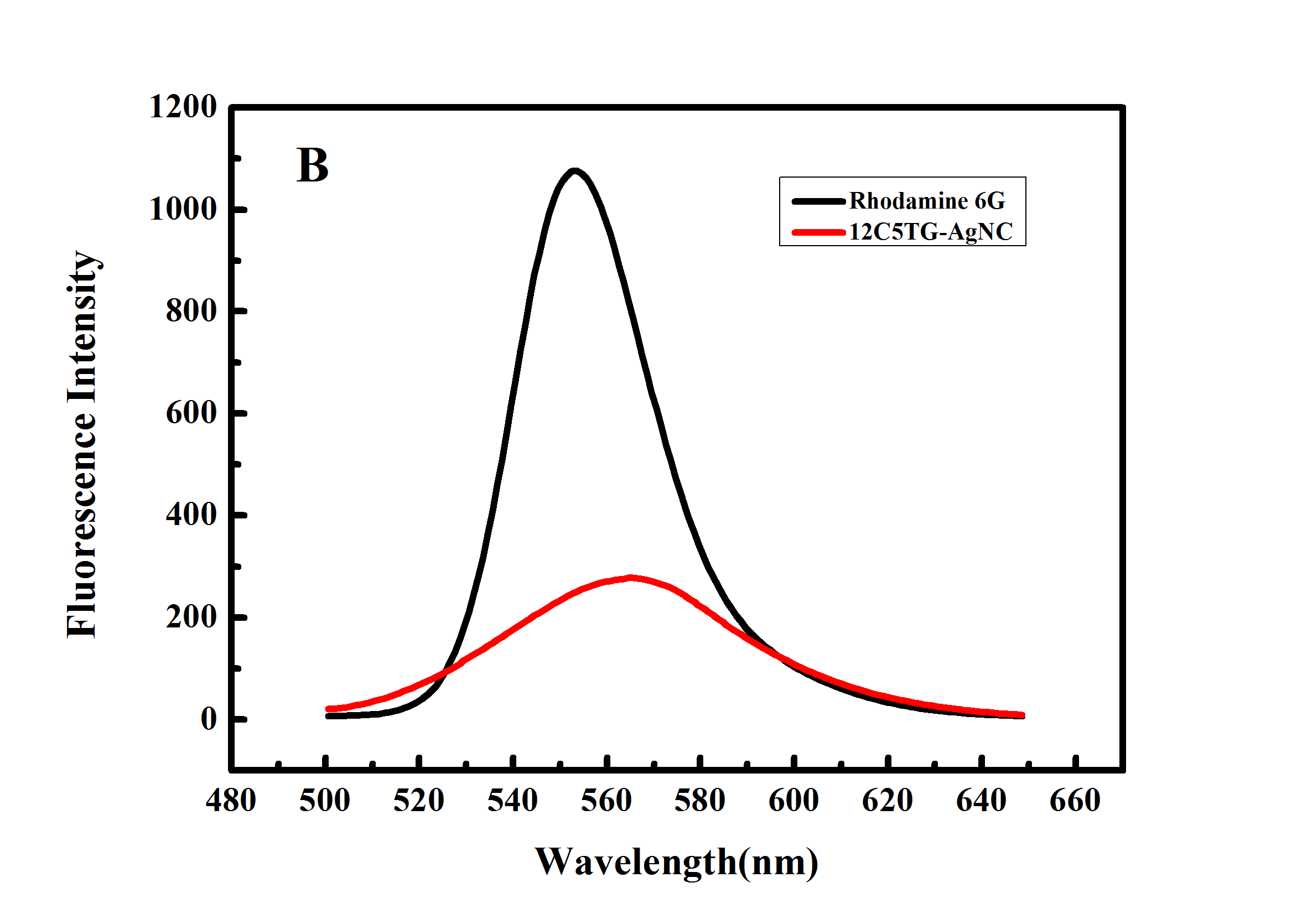
**

**Figure S1.** Quantum yield of 12C5TG-AgNC. (A) Absorption spectra of standard Rhodamine 6G (black) and 12C5TG-AgNC (red); (B) Their fluorescence emission spectra with excitation wavelength of 498 nm, where the absorbance values of them are equal.

Chrysin Daidzein Quercetin

Emodin Aloe-emodin Kaempferol

Chrysophanic acid Apigenin Luteolin

AED3 Matrine Colchicine

**Figure S2*.*** The chemical structural formulas of ligands used in the assay.

**
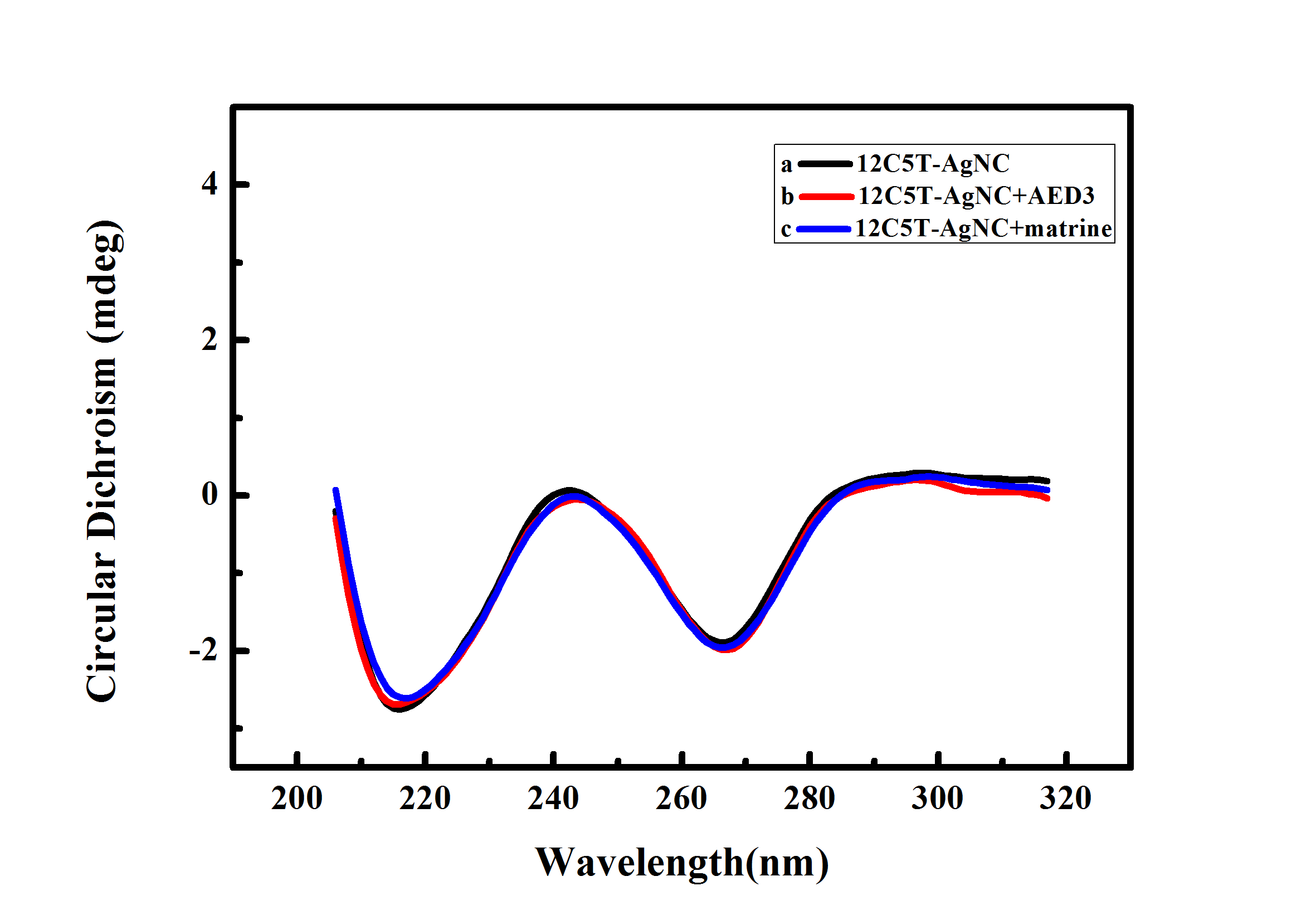
**

**Figure S3*.*** CD spectra of (a) 2.0 µM 12C5T-AgNC, (b) (a)+20.0 µM AED3, (c) (a)+20.0 µM matrine.


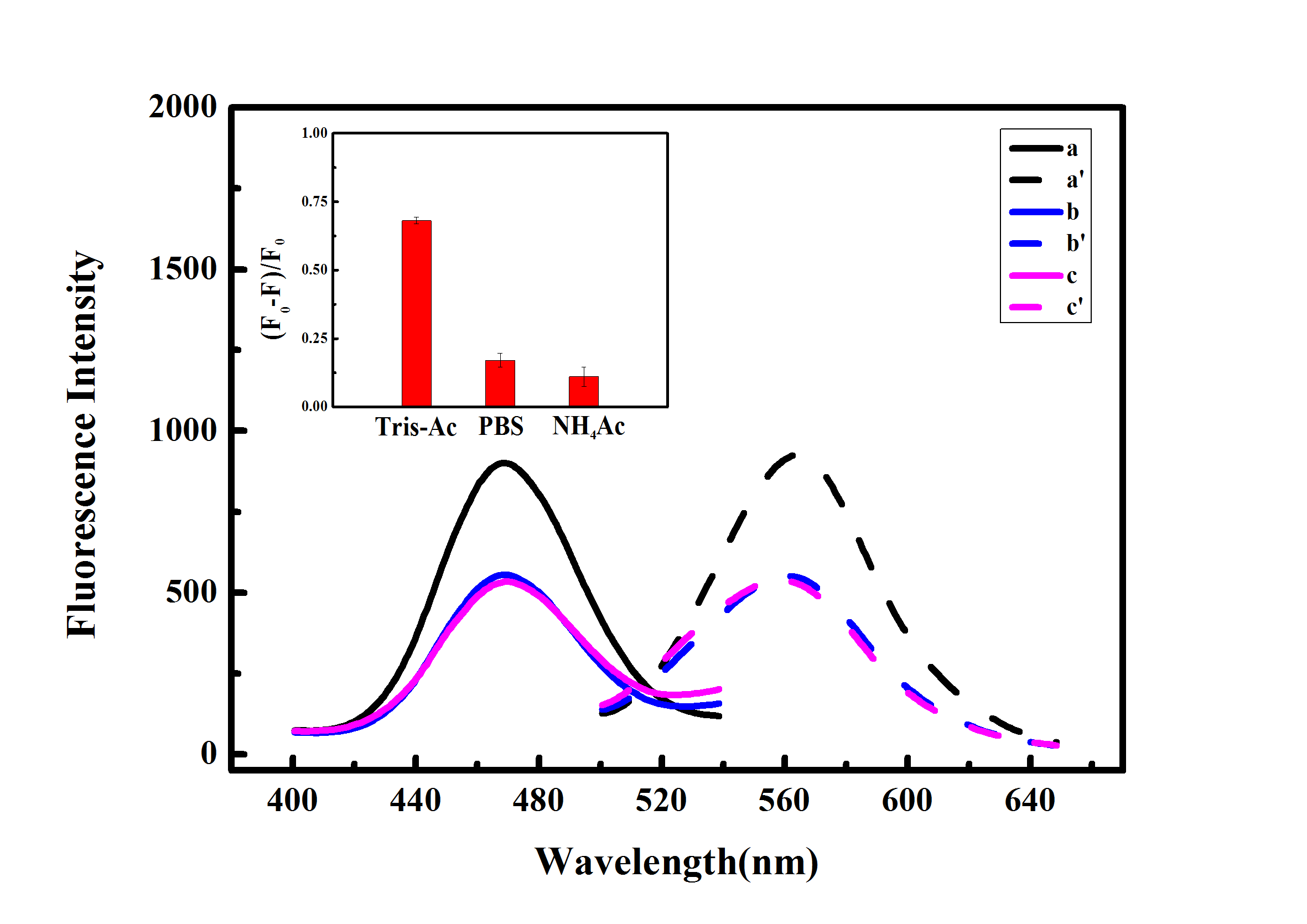


**Figure S4*.*** Fluorescence excitation/emission spectra of 1.0 µM 12C5TG-AgNC in (a/a′) 10 mM Tris-Ac buffer, (b/b′) 20 mM PBS buffer, (c/c′) 40 mM ammonium acetate buffer. Inset is fluorescence quenching efficiency (F_0_-F)/F_0_, where F and F_0_ were the fluorescence intensity of 1.0 µM AgNC in the presence and absence of 10.0 µM AED3, respectively.


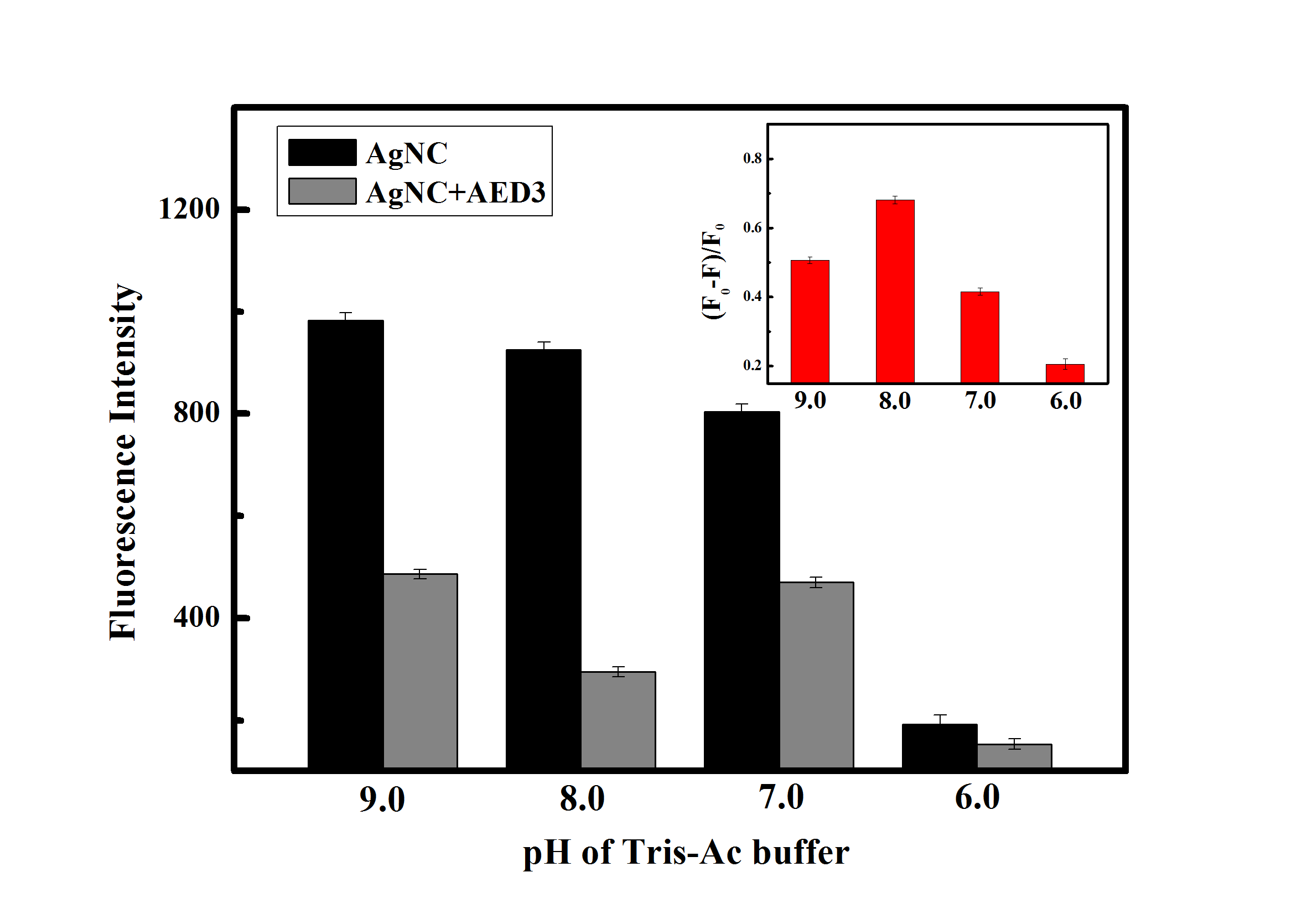


**Figure S5*.*** Fluorescence intensity of 1.0 µM 12C5TG-AgNC with 10.0 µM AED3 in Tris-Ac buffer of different pH: 9.0/8.0/7.0/6.0. Inset is fluorescence quenching efficiency (F_0_-F)/F_0_, where F and F_0_ were the fluorescence intensity of 1.0 µM AgNC in the presence and absence of 10.0 µM AED3, respectively.


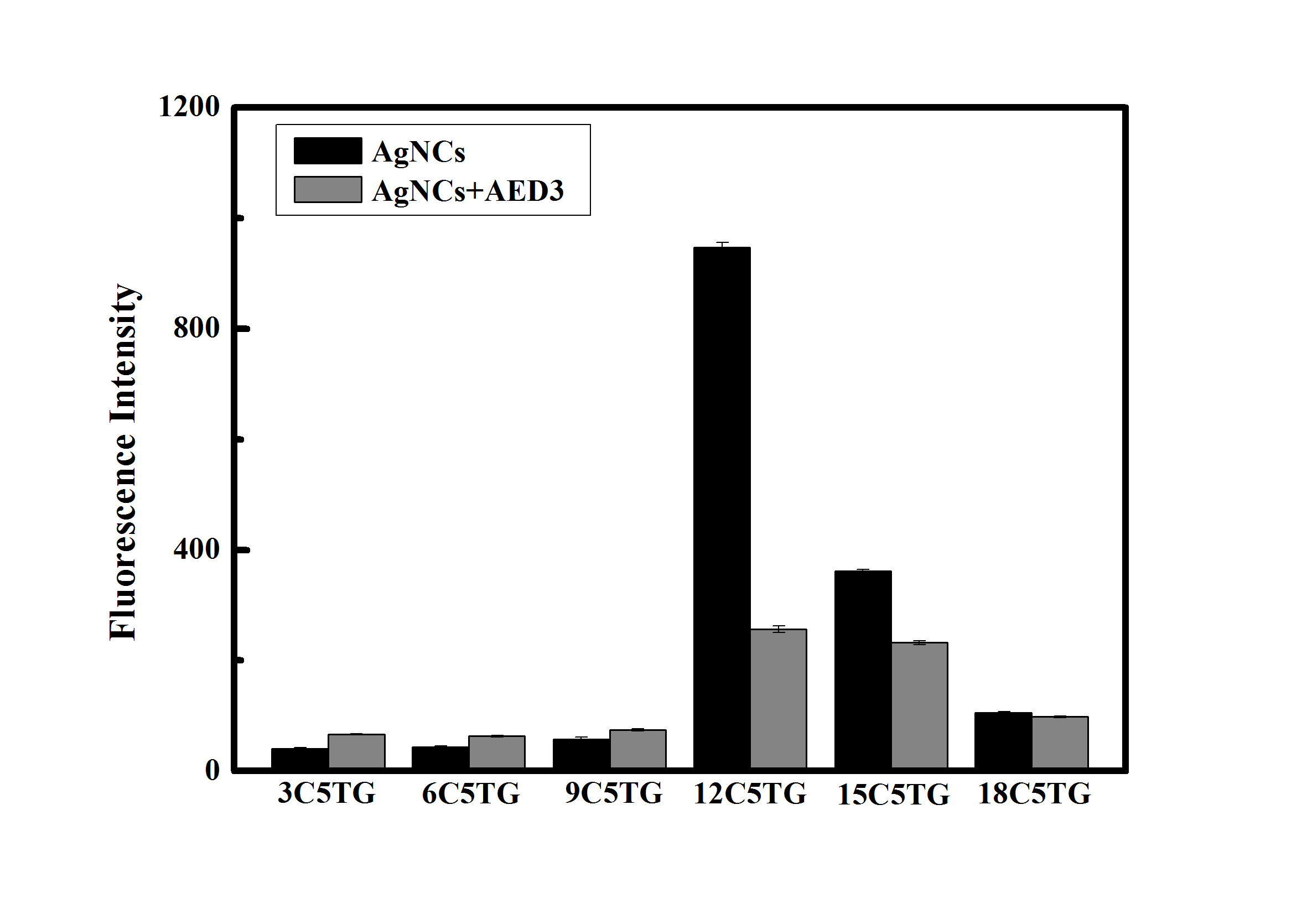


**Figure S6*.*** Fluorescence intensity of 1.0 µM nC5TG-templated AgNC with 10.0 µM AED3, where n represents the different numbers of cytosine nucleotide (3, 6, 9, 12, 15, 18).


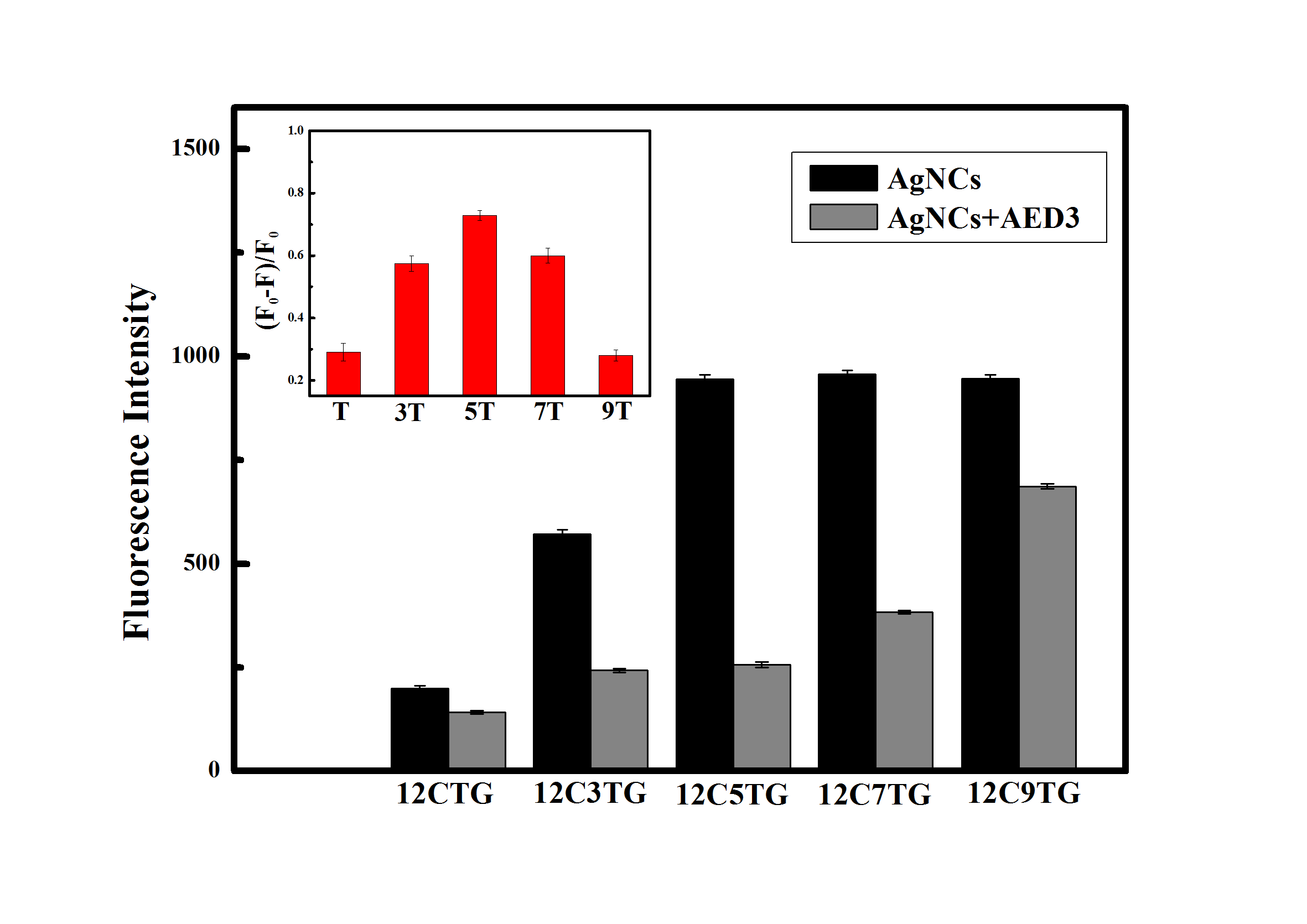


**Figure S7.** Fluorescence intensity of 1.0 µM 12CnTG-templated AgNC with 10.0 µM AED3, where n represents the different numbers of thymine nucleotide (1, 3, 5, 7. 9). Inset is fluorescence quenching efficiency (F_0_-F)/F_0_, where F and F_0_ were the fluorescence intensity of 1.0 µM AgNC in the presence and absence of 10.0 µM AED3, respectively.

#
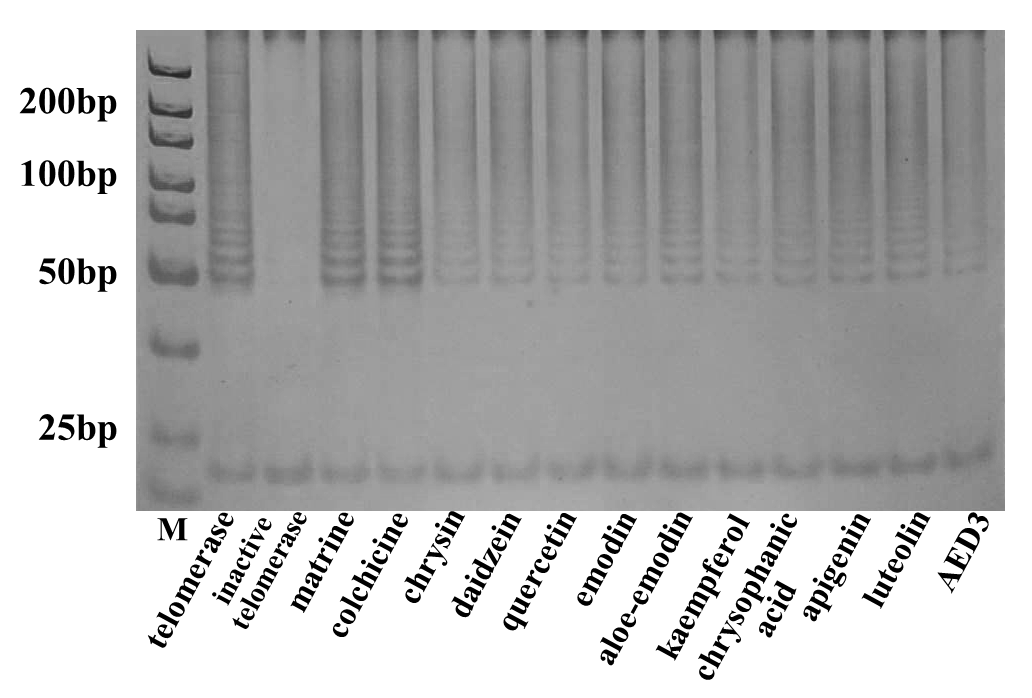


**Figure S8.** Evaluation of telomerase activity by TRAP assay. The concentration of ligands is 50 µM. There are 500 HeLa cells..

#
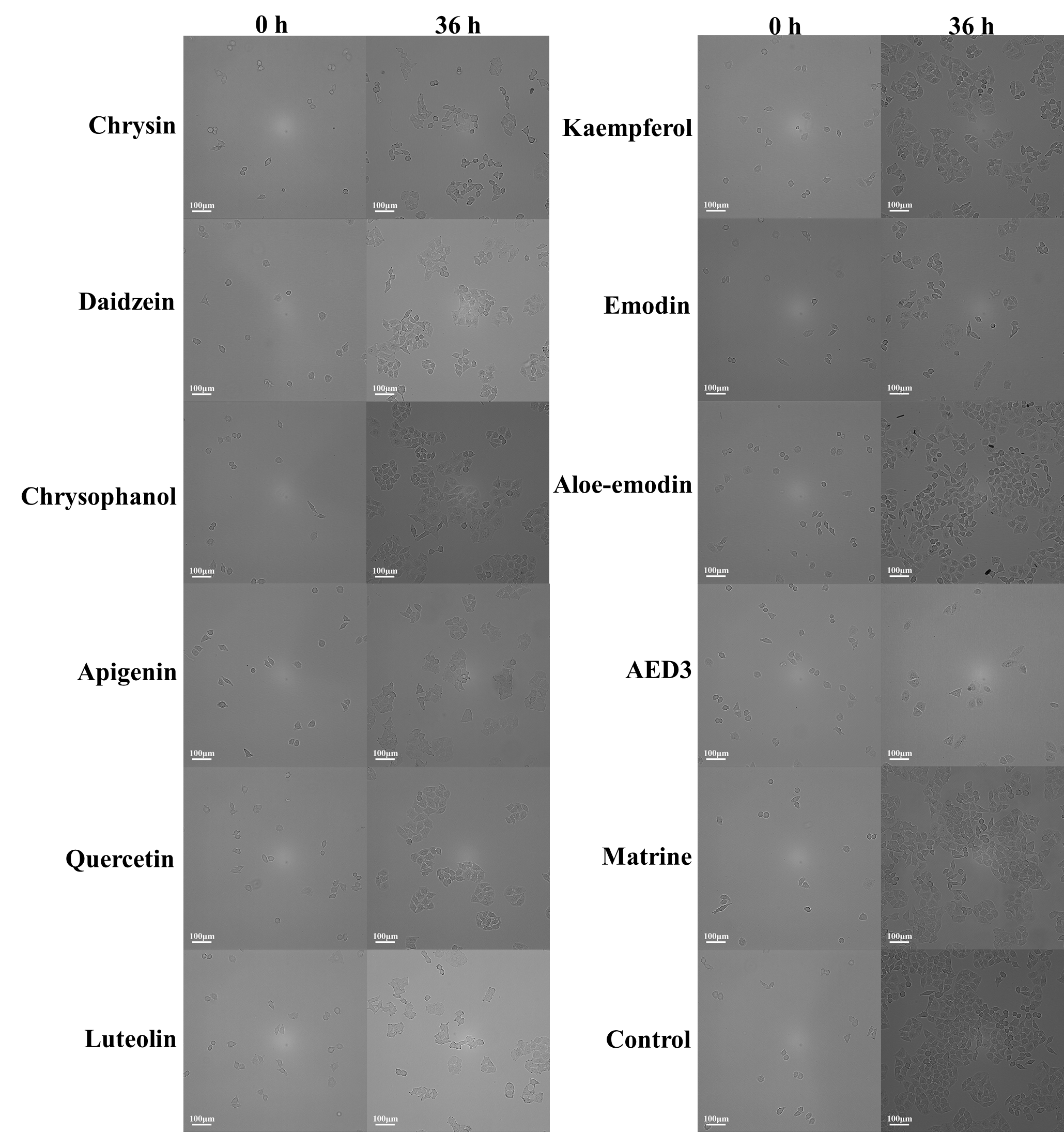


**Figure S9.** Time course of images of HeLa incubated with ligands.


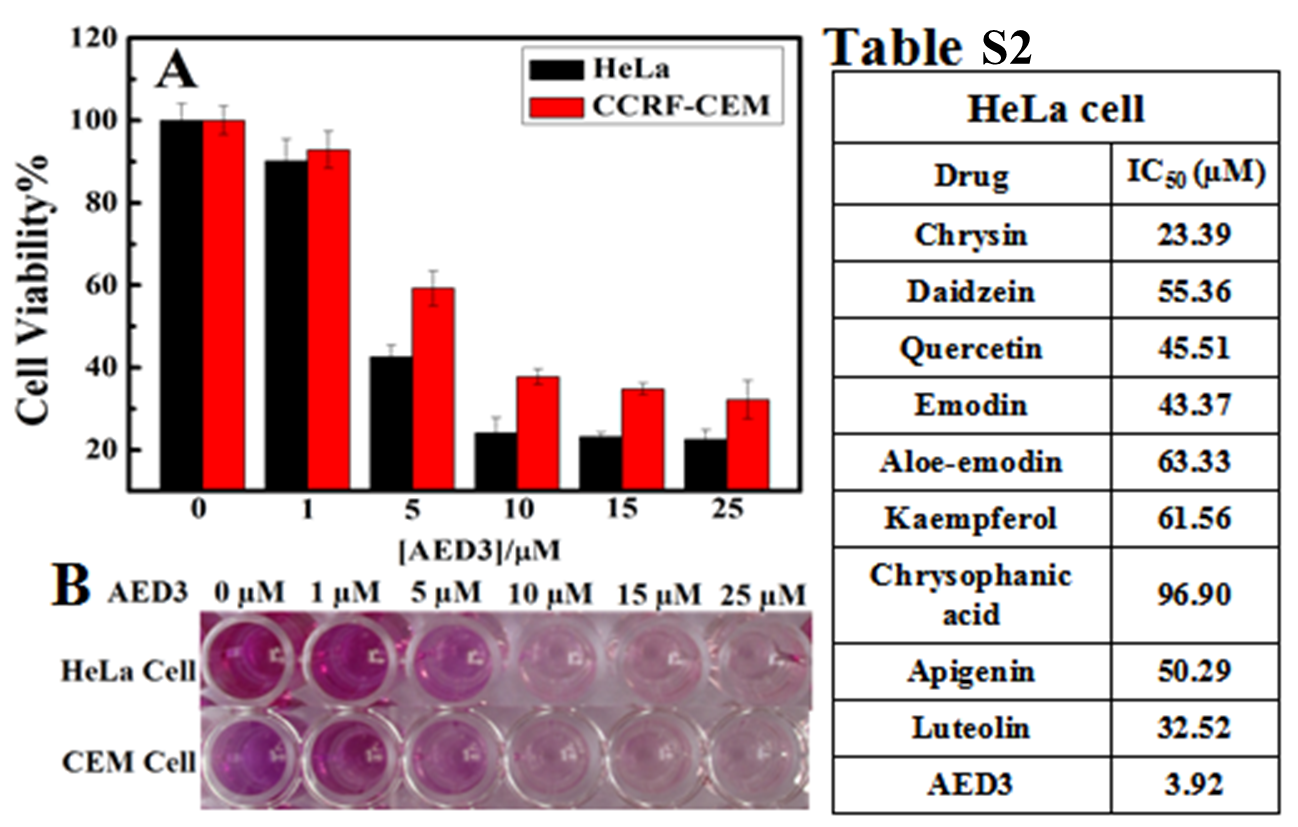


**Figure S10.** (A) Histogram comparison of Cell Viability% of HeLa and CCRF-CEM which were incubated with different concentrations of AED3 for 48 h. Error bars were calculated from three replicate measurements.(B) MTT photographs of HeLa and CCRF-CEM. The experiment was performed in 96-well plate. Table S2 is inhibition effects (IC_50_) of all the selected ligands on HeLa after incubated for 48 h.
